# Suspected CNS Metastases of Askin's Tumor: Would You Irradiate the Neural Axis?

**DOI:** 10.4021/wjon564w

**Published:** 2013-01-04

**Authors:** Orit Kaidar-Person, Ayelet Eran, Nissim Haim, Yaakov Amsalem, Abraham Kuten, Gil Bar-Sela

**Affiliations:** a Division of Oncology, Rambam Health Care Campus, Haifa, Israel; b Department of Radiology, Rambam Health Care Campus, Haifa, Israel

**Keywords:** Askin’s tumor, CNS metastases

## Abstract

We present a case of a young-adult patient who was diagnosed with Askin’s tumor, with central nervous system lesions suspected as metastases. The patient achieved complete response after chemotherapy, and the question of consolidation radiotherapy to the CNS is discussed.

## Introduction

Brain metastases from sarcomas are relatively uncommon and data are limited. The management of brain metastatic sarcoma patients is challenging since these lesions are often refractory to treatment and there are no currently accepted guidelines or consensus of the role of surgery, chemotherapy and radiation in the treatment of brain metastasis [1-4]. Peripheral primitive neuroectodermal tumor (PNET) is a soft tissue sarcoma, described as arising intracranially [5-7]. We present a case of adult-onset Askin’s tumor (thoracopulmonary PNET) with central nervous system lesions suspected as metastases.

## Case Report

**Figure 1:**
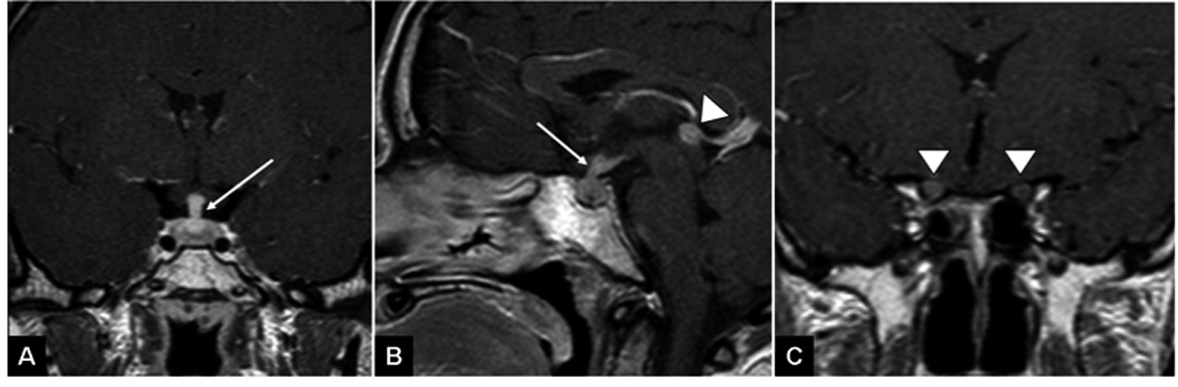
T1 weighted images following gadolinium injection demonstrate thickened pituitary infundibulum (arrow A, B), thickened and enhancing optic nerves (arrowheads C) and a nodular enhancing pineal lesion (arrowhead B).

**Figure 2:**
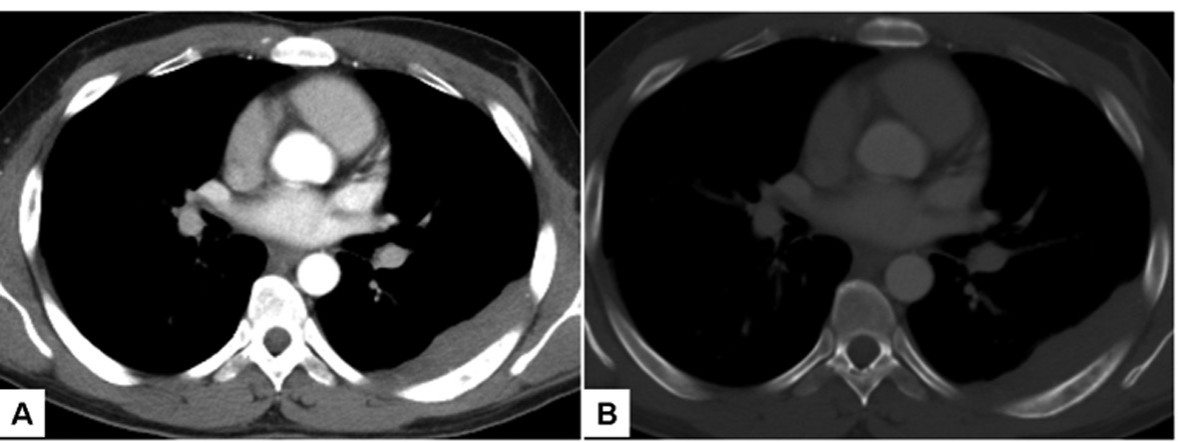
Axial CT images through mid-thorax demonstrate pleural-based soft tissue mass along the posterior aspect of the left hemithorax (A). On bone window (B), permeative destruction of the adjacent 6th rib is noted.

The patient was a 28-year-old male from Jewish-Ethiopian descent, with no history of chronic diseases. He presented with right eye pain lasting for three weeks and loss of vision in the same eye. Fundoscopy showed bilateral papilledema and unknown cells within the vitreous. A brain MRI showed enhancement of the pituitary infundibulum and thickening of the optic nerves and optic chiasm, as well as an enhancing lesion in the pineal gland (Fig. 1). Whole body CT scan showed a left sided chest wall lesion with destruction of the 6th rib (Fig. 2). Tumor markers, including beta-HCG, alpha-FP, were within the normal limits. Since the differential diagnosis of the CNS lesions included sarcoid, angiotensin and calcium levels in the blood and CSF were examined and found to be within the normal limits. Repeated CSF examination showed normal biochemistry, and cytotyping of lymphocytes from the lumbar puncture was compatible with reactive lymphocytes; malignant cells were not demonstrated. CSF was negative for CMV DNA, Ig for West Nile virus, HHV-6 DNA, HSV-1 DNA, Varicella Zoster DNA. A core needle biopsy was taken from the pleural lesion and was consistent with poorly differentiated PNET with negative FISH and PCR for translucation (t 11; 22). The tumor cells stained positively via immunohistochemistry for synaptophysin and CD-56, but were negative for LCA, CD-20, CD-3, CD-43, TdT, PAX-5; vimentin, FLI-1; cytokeratin, Cytokeratin-7, Cytokeratin -20, P-63, and inconclusive for chromogranin. Bone marrow aspiration was normal without evidence of malignant cells. The possibility of taking a biopsy or cytology from the brain lesion or the vitreous was ruled out by the neurosurgeons and ophthalmologists in our institution, due to the high surgical risk.

On presentation, the patient was started on high doses of steroids with immediate improvement of his eyesight. After histological evidence of PNET, chemotherapy was started with etoposide-ifosfamide alternating with vincristine, doxorubicin and cyclophosphamide cycles. A brain MRI on the 4th day of the first chemotherapy cycle (and after two weeks of steroid treatment) revealed disappearance of the lesion in the pineal gland and partial resolution of the other CNS sites. The chest wall lesion regressed significantly after chemotherapy.

After the 4th chemotherapy cycle the patient underwent thoracotomy with resection of the chest wall lesion; pathological examination revealed complete remission. The patient completed a year of chemotherapy treatment as noted above.

## Discussion

In a retrospective study of CNS involvement in children with sarcoma, 11 of 19 patients included in the study had Ewing's sarcoma [8]. Various treatment combinations were applied according to the patient's medical status: chemotherapy alone (4/19), radiotherapy (2/19), surgery (1/19), surgery and subsequent chemotherapy (1/19), chemotherapy and radiotherapy (7/19), and three patients received best supportive care. Regardless of treatment, most patients died of brain disease and the mean duration from the time of diagnosis of CNS involvement to time of death was five months. The authors reported that there were no significant differences between treatments [8].

Consolidation radiotherapy following a chemotherapy response in a young patient with otherwise good performance status, oligometastatic disease, and controlled primary site is often the treatment of choice. If the reported patient actually had involvement of the vitreous and intracranial disease, craniospinal irradiation (CSI) and orbital irradiation may be indicated due to the risk of leptomeningeal dissemination. CSI is technically challenging because of the planning target volume (PTV) length and the vital organs exposed. Nowadays, innovative techniques, such as three-dimensional conformal radiotherapy, proton beam, intensity modulated techniques, and tomotherapy, can be applied with great precision [9, 10]. However, this patient had such a rapid improvement in the CNS with steroids, this might indicate that two extraordinarily rare events occurred in one person simultaneously: Askin's tumor of the rib and a different diagnosis in the CNS (such as CNS lymphoma, Langerhans’ cell histiocytosis, or CNS sarcoidosis) [11, 12]. It is hard to accept the toxicity of such treatments when the patient has responded so well to steroids and chemotherapy and when there is a slight chance that the CNS lesion is not a metastasis. Moreover, as reported by Postovsky et al [8], there was no significant difference between patients who received direct CNS therapy and those who received chemotherapy or best supportive care.

After consulting with a highly regarded overseas center, it was decided not to irradiate the CNS and to follow with brain MRI and ophthalmological examination every three months. An evaluation performed six months after completion of treatment indicated that the patient's eyesight was stable, his eye exam was unremarkable, and there was no evidence of chest wall recurrence and or of CNS disease on brain MRI.

The presented case raises the following dilemmas: How much to risk a patient to obtain a tissue diagnosis from the intracranial lesion? Assuming the lesions were metastases, is consolidation treatment after complete response necessary? Most importantly and finally, should the craniospinal axis be irradiated?
